# Association of Childhood Fat Mass and Weight With Adult-Onset Type 2 Diabetes in Denmark

**DOI:** 10.1001/jamanetworkopen.2021.8524

**Published:** 2021-04-30

**Authors:** Mohammed T. Hudda, Julie Aarestrup, Christopher G. Owen, Derek G. Cook, Thorkild I. A. Sørensen, Alicja R. Rudnicka, Jennifer L. Baker, Peter H. Whincup, Claire M. Nightingale

**Affiliations:** 1 Population Health Research Institute, St George’s, University of London, Cranmer Terrace, London, United Kingdom; 2 Center for Clinical Research and Prevention, Bispebjerg and Frederiksberg Hospital, The Capital Region, Copenhagen, Denmark; 3 Department of Public Health, University of Copenhagen, Copenhagen, Denmark; 4 Novo Nordisk Foundation Center for Basic Metabolic Research, University of Copenhagen, Copenhagen, Denmark

## Abstract

**Importance:**

Childhood obesity, defined by cutoffs based on the weight-based marker of body mass index, is associated with adult type 2 diabetes (T2D) risk. Whether childhood fat mass (FM) is the driver of these associations is currently unknown.

**Objective:**

To quantify and compare height-independent associations between childhood FM and weight with adult T2D risk in a historic Danish cohort.

**Design, Setting, and Participants:**

This population-based retrospective cohort study included schoolchildren from The Copenhagen School Health Records Register born between January 1930 and December 1985 with follow-up to adulthood through December 31, 2015. Analyses were based on 269 913 schoolchildren aged 10 years with 21 896 established adult T2D cases and 261 192 children aged 13 years with 21 530 established adult T2D cases for whom childhood height and weight measurements, as well as predicted FM, were available. Statistical analyses were performed between April 2019 to August 2020.

**Exposures:**

Childhood FM and weight at ages 10 and 13 years.

**Main Outcomes and Measures:**

Diagnoses of T2D were established by linkage to national disease registers for adults aged at least 30 years. Sex-specific Cox regression quantified associations, adjusted for childhood height, which were evaluated within 5 birth-cohort groups. Group-specific results were pooled using random-effects meta-analyses accounting for heterogeneity across group-specific associations.

**Results:**

This cohort study analyzed data from 269 913 children aged 10 years (135 940 boys [50.4%]) with 21 896 established adult T2D cases and 261 192 children aged 13 years (131 025 boys [50.2%]) with 21 530 established adult T2D cases. After adjusting for childhood height, increases in FM and weight (per kilogram) among boys aged 10 years were associated with elevated T2D risks at age 50 years of 12% (hazard ratio [HR], 1.12; 95% CI, 1.10-1.14) and 7% (HR, 1.07; 95% CI, 1.05-1.09), respectively, and among girls aged 10 years of 15% (HR, 1.15; 95% CI, 1.13-1.17) and 10% (HR, 1.10; 95% CI, 1.08-1.11), respectively. Among children aged 13 years, increases in FM and weight (per kilogram) were associated with increased T2D risks at age 50 years of 10% (HR, 1.10; 95% CI, 1.09-1.10) and 6% (HR, 1.06; 95% CI, 1.05-1.07) for boys, respectively, and of 10% (HR, 1.10; 95% CI, 1.10-1.11) and 7% (HR, 1.07; 95% CI, 1.06-1.08), respectively, for girls.

**Conclusions and Relevance:**

This cohort study found that a 1-kg increase in childhood FM was more strongly associated with increased adult T2D risk than a 1-kg increase in weight, independent of childhood height. Information on FM, rather than weight-based measures, focuses on a modifiable component of weight that may be associated with adult T2D risk. These findings support the assessment of childhood FM in adiposity surveillance initiatives in an effort to reduce long-term T2D risk.

## Key Points

**Question:**

Is childhood fat mass (FM) more strongly associated with long-term type 2 diabetes (T2D) risk than childhood weight, independent of height?

**Findings:**

In a population-based cohort study including more than 260 000 schoolchildren in Denmark, there were stronger associations (per kilogram increase) between childhood FM and adult T2D risk, independent of childhood height, than those observed for childhood weight.

**Meaning:**

Results suggest that weight-based measures, currently used as the basis for childhood obesity assessment in the form of body mass index, were less strongly associated with adult T2D risk than childhood FM.

## Teaser

This cohort study uses data from Copenhagen School Health Records Register to investigate the association of childhood fat mass and weight with adult-onset type 2 diabetes.

## Introduction

Obesity is a substantial global public health issue in both childhood and adulthood. The World Health Organization estimated that in 2016 there were more than 340 million children and adolescents aged 5 to 19^1^ years worldwide affected by overweight (including obesity) and a further 41 million younger than age 5 years.^1^ Overweight is a strong determinant of type 2 diabetes (T2D) risk; this association is apparent for children who are overweight^2^ as well as adults.^3,4^ Such reported associations have generally been based on childhood body mass index (BMI), a weight-for-height measure, which is widely used in clinical and public health practice. However, BMI has a number of limitations as a marker of body fatness (BF) in children,^5,6,7^ particularly its inability as a weight-based measure to discriminate between lean mass (fat-free mass [FFM]) and fat mass (FM), the balance of which can vary markedly in individuals with a given BMI.^6^ BMI is only moderately associated with childhood BF^8,9^ and is correlated with childhood height.^8,10^ Although it has been suggested that this correlation might reflect higher levels of BF in taller children,^11,12^ short stature has been shown to be independently associated with adult T2D risk.^13^ It would be of considerable importance to establish whether childhood FM per se is more strongly associated with T2D risk than childhood weight-based measures, independent of height. Although previous studies in children and adolescents have shown significant cross-sectional associations between FM and cardiometabolic risk factors,^14,15^ which have been of comparable magnitude to those of BMI,^14^ there is little or no information, to our knowledge, on the long-term associations between fat-based measures in childhood and their associations with incident T2D in adulthood. This lack of data reflects the limited number of long-term cohort studies with information on childhood FM as well as established adult T2D diagnoses. However, the recently developed and extensively validated prediction equation for estimating FM based on height, weight, and the demographic factors of age, sex, and race/ethnicity^16^ allows for retrospective BF estimation within historic data sets with established information on disease outcomes in adulthood, including T2D. Therefore, we examined the height-independent associations between childhood FM and weight with adult T2D risk among a large Danish cohort of schoolchildren born between 1930 and 1985, measured in childhood and followed up for T2D incidence in adulthood.^17^

## Methods

### Study Population

This population-based cohort study was based on The Copenhagen School Health Records Register (CSHRR), a database containing information on almost every schoolchild in Copenhagen born between January 1930 and December 1985.^17^ For legal and procedural reasons, there were variations in the ages at which children in the cohort were measured throughout the years. The cohort members had mandatory health examinations, including height and weight measurements, which were recorded by school-based doctors or nurses.^17^

The Danish Data Protection Agency approved this study under The Capital Region, Denmark permission No. 2007-58-0015. According to Danish law, purely register-based studies in which individuals are not contacted are exempt from approval by an ethics committee. This was a mandated program from the Danish government and therefore all children were required to participate. This study followed the Strengthening the Reporting of Observational Studies in Epidemiology (STROBE) reporting guideline.

Using individual personal identification numbers, which are issued to Danish citizens alive or born after 1968, data from the CSHRR were linked with the Danish National Patient Register (NPR) to obtain inpatient and outpatient diagnoses of T2D. The NPR contains hospital discharge diagnoses from all hospitals since 1977 and from outpatient and emergency departments since 1995.^18^ The date of the first hospital registration was used to define the age at diagnosis. As the NPR only includes individuals who were referred to hospital-based clinics (and not patients who are diagnosed with T2D in general practice), the total population-level, age-specific incidence of T2D was underestimated in this study. However, as T2D in Denmark is managed using a “shared care” model, general practitioners often refer patients to treatment at specialized hospital outpatient clinics so that even individuals with typical diabetes are frequently treated in a hospital setting and would be included within the NPR. As a result, the extent of underestimation, particularly with extended follow-up duration, is likely to be limited. The *International Classification of Diseases, Eighth Revision (ICD-8)* until 1994 (code 250) and the *International Statistical Classification of Diseases and Related Health Problems, Tenth Revision (ICD-10)* thereafter (codes E11 through E14) were used to define T2D. In 1987, code 249 (insulin-dependent diabetes mellitus) was introduced in Denmark; previously, code 250 had included all forms of diabetes. To minimize the potential for misclassification, cases of insulin-dependent diabetes mellitus and diabetes diagnosed before age 30 years were excluded because type 1 diabetes is insulin-dependent and generally tends to be diagnosed at earlier ages.^19^

Of 316 340 individuals potentially eligible for this study, 11 282 emigrated, died, or were untraceable before the initiation of follow-up on January 1, 1977, or age 30 years (eFigure in the Supplement). A total of 827 individuals diagnosed with diabetes before age 30 years or the start of follow-up were excluded, together with 5106 individuals missing childhood anthropometric data and 7 individuals with outlying values at all childhood ages (eFigure in the Supplement). This study included all individuals with complete information on height, weight, age, sex, and date of the health examination for at least 1 childhood measurement at age 7, 10, or 13 years and who were alive at the start of the diabetes follow-up period at age 30 years. Follow-up ended on the date of a T2D diagnosis, death, emigration, loss to follow-up, or December 31, 2015, whichever was first.

### Statistical Analysis

#### Prediction of Childhood FM

Statistical analyses were performed from April 2019 to August 2020 using Stata, version 15 (StataCorp LLC). The equation used to estimate childhood FFM and FM from height, weight, age, and sex for this study was derived in a data set of 2375 UK children aged 4 to 15 years who had reference standard deuterium dilution measures of body composition.^16^ The equation was extensively validated both internally (to assess model overfitting) and externally (to assess model generalizability). External validation in UK children aged 11 to 12 years demonstrated promising model generalizability (*R^2^* = 90.0%) and good calibration of observed and predicted FM (calibration slope, 1.02; 95% CI, 0.97-1.07) with a mean difference between observed and predicted FM of −1.29 kg.^16^ A comparison of the FM predictions, obtained from the model with those obtained from dual-energy x-ray absorptiometry and bioelectrical impedance, found this equation to provide FM estimates at least as accurate as those of dual-energy x-ray absorptiometry and bioelectrical impedance.^20^ A supplement file containing a Microsoft Excel–based calculator was published alongside the original publication, to facilitate the quick and straightforward estimation of FFM and FM using this equation. This equation was used to estimate FFM (and FM by subtraction from weight measurement) within the CSHRR. Information on race/ethnicity was not available but was assumed to be White for all individuals, as the proportion of children from a non-White European ethnic background was likely to be low owing to migration patterns to Denmark at this time.^17^ Correlation coefficients were calculated between childhood FM, FFM, weight, and height. Childhood measurements were also summarized using mean and SD, by adult T2D case status, and birth-year cohort group within the study population.

#### Associations Between Childhood FM and Weight With T2D

Cox proportional-hazards regression models were used to examine the respective associations between childhood FM and weight with adult T2D risk. The underlying timescale of the Cox models was age, with follow-up beginning at age 30 years. At each childhood age of 7, 10, and 13 years, sex-specific models were fitted for each of the 2 childhood exposures (FM and weight) and adjusted for childhood height (as a continuous variable), a potential confounder of the associations of interest. To account for potential heterogeneity in associations across the wide range of birth years, these models were also all fitted separately within 5 birth-cohort groups (1930-1939, 1940-1949, 1950-1959, 1960-1969, and 1970-1985).

#### Model Assumptions

The linearity assumption within the sex- and birth-cohort–specific Cox models described previously (ie, assuming a log-linear association between the exposures of interest [FM and weight] at each childhood age and adult T2D risk) was tested by refitting each Cox model and including a nonlinear restricted cubic spline term with 3 knots for the childhood exposure of interest (either FM or weight) as well as for height. The likelihood ratio test was used to determine the statistical significance (*P* < .05) of the nonlinear cubic spline terms. Results of tests for the significance of cubic spline terms (data not presented) suggested largely log-linear patterns at both 10 and 13 years for both FM and weight associations, but not at 7 years. Therefore, analyses in this study focused on quantifying the FM and weight associations at 10 and 13 years of age. The proportional-hazards assumption was assessed by testing the statistical significance (Wald test at the 5% significance level) of time-varying coefficients and interaction terms between adult age and the main exposure (FM or weight) within each of the fitted Cox models. There was evidence to suggest that the assumption was violated (ie, associations between childhood FM or weight and T2D risk were not constant across the range of adult ages); therefore, time-varying coefficients were retained within final sex- and birth-cohort–specific models.

Hazard ratios (HRs) and 95% CIs were estimated from the Cox models, adjusting for childhood height (as a continuous variable) and including time-varying coefficients for a range of adult ages, including 30, 40, 50, 60, and 70 years. This was done for each birth-cohort group without extrapolation beyond the greatest adult age in the birth-cohort group; hence, associations at older adult ages were not quantified for more recent birth-cohort groups in which individuals had not yet reached those ages. Owing to the low levels of meaningful heterogeneity between associations from birth-cohort groups at given ages, HRs from each of the 5 birth-cohort groups were averaged using random-effects meta-analysis to provide a pooled estimate of the associations of interest across birth-cohort groups for each age group. As FM and weight are measured on the same scale, we presented the effect sizes per-kilogram increase in the childhood adiposity markers. Effect sizes were also presented per SD increase in exposure variables. However, because weight can be partitioned into FM and FFM, and it follows by definition that the SD of weight is a combination of the SDs of FM and FFM, comparisons on the SD scale of effect sizes for weight with those of FM should be interpreted with caution because they provide a different perspective on the effect of childhood body composition on long-term T2D risk. As the SDs of FM and weight differed by birth-cohort group, the SDs used to transform the effect sizes onto the SD scale were calculated within birth-cohort; therefore, results were not pooled across groups as was done for the per-kilogram analysis.

## Results

Childhood measurements of weight, height, and predicted FM and FFM contained no missing data and were available across the 5 birth-cohort groups in 269 913 children aged 10 years (135 940 boys [50.4%] and 133 973 girls [49.6%]) and in 261 192 children aged 13 years (131 025 boys [50.2%] and 130 167 girls [49.8%]). Mean levels and SDs for each variable at both 10 and 13 years of age in boys and girls are presented by adult T2D diagnosis status in Table 1 and Table 2 and overall in eTable 1 in the Supplement. Mean levels of each variable increased in more recent birth cohorts at both 10 and 13 years of age (eTable 1 in the Supplement). At 10 years of age, across all birth-cohort groups, the mean (SD) weight was similar among girls (31.6 [5.3] kg) and boys (31.9 [4.9] kg), as was height (girls, 1.37 [0.06] m; boys, 1.38 [0.06] m), but girls had higher mean (SD) levels of FM (8.2 [2.7] kg) compared with boys (7.1 [2.4] kg) (eTable 1 in the Supplement). Although mean childhood height was similar among those with future T2D compared with those who did not develop T2D, average FM and weight levels were higher among those who went on to develop T2D vs those who did not (eg, boys, 10 y overall, mean [SD] weight, 32.0 [5.1] kg and FM, 7.3 [2.5] kg vs weight, 31.8 [4.8] kg and FM, 7.1 [2.4] kg; boys, 13 y overall, weight, 43.8 [8.3] kg and FM, 9.6 [3.9] kg vs weight, 43.0 [7.8] kg and FM, 8.8 [3.5] kg) (Tables 1 and 2). This difference, which was observed at ages 10 and 13 years among both sexes, was more marked in more recent birth-cohort groups. Furthermore, the percentage of weight attributed to FM was greater in more recent birth-cohort groups than in earlier groups (eg, boys 10 years overall, born 1970-1985, average FM as percentage of average weight = 23.6% vs 21.7% for boys, 10 years overall, born 1930-1939) (Tables 1 and 2; eTable 1 in the Supplement). A significant association was observed between height and both FFM and weight in childhood at 10 and 13 years of age, whereas FM was only moderately associated with childhood height at 10 and 13 years of age (10 years, height and FFM, *r* = 0.9 boys and girls; height and weight, *r* = 0.8 for boys and *r* = 0.7 for girls; 13 years, height and FFM, *r* = 0.9 boys and girls; height and weight, *r* = 0.8 for boys and *r* = 0.7 for girls), whereas FM was only moderately associated with childhood height (10 years, height and FM, *r* = 0.5 boys and girls; 13 years, height and FM, *r* = 0.4 boys and girls) (eTable 2 in the Supplement).

**Table 1.** Weight, Fat Mass, Fat-Free Mass, and Height Among Boys by Adult Type 2 Diabetes Status and Birth-Cohort Group

Birth-year cohort group	10 y					13 y				
	No. (%)	Mean (SD)				No. (%)	Mean (SD)			
		Weight, kg	Fat mass, kg	Fat-free mass, kg	Height, m		Weight, kg	Fat mass, kg	Fat-free mass, kg	Height, m
**Those who developed type 2 diabetes**										
1930-1939	4588 (34.6)	30.6 (4.2)	6.7 (1.9)	23.9 (2.5)	1.36 (0.06)	4410 (33.9)	41.5 (7.0)	8.7 (3.2)	32.8 (4.4)	1.51 (0.07)
1940-1949	5554 (41.9)	32.3 (4.9)	7.5 (2.5)	24.9 (2.8)	1.38 (0.06)	5508 (42.4)	44.3 (8.0)	9.7 (3.8)	34.6 (4.9)	1.54 (0.08)
1950-1959	2317 (17.5)	33.0 (5.5)	7.7 (2.9)	25.2 (2.9)	1.39 (0.06)	2299 (17.7)	45.7 (8.9)	10.2 (4.5)	35.5 (5.2)	1.55 (0.08)
1960-1969	684 (5.2)	34.4 (6.2)	8.4 (3.3)	26.1 (3.3)	1.40 (0.06)	686 (5.3)	48.1 (9.6)	11.1 (4.9)	37.0 (5.7)	1.57 (0.09)
1970-1985	119 (0.9)	37.5 (9.4)	10.4 (5.4)	27.2 (4.3)	1.41 (0.07)	97 (0.7)	52.8 (13.2)	13.8 (7.0)	39.0 (7.3)	1.58 (0.09)
Overall	13 262 (100)	32.0 (5.1)	7.3 (2.5)	24.7 (2.8)	1.38 (0.06)	13 000 (100)	43.8 (8.3)	9.6 (3.9)	34.3 (5.0)	1.53 (0.08)
**Those who did not develop type 2 diabetes**										
1930-1939	25 329 (20.1)	30.4 (3.9)	6.6 (1.8)	23.8 (2.4)	1.36 (0.06)	24 345 (20.6)	40.7 (6.4)	8.2 (2.8)	32.4 (4.2)	1.51 (0.07)
1940-1949	38 265 (31.2)	31.8 (4.5)	7.1 (2.2)	24.7 (2.6)	1.38 (0.06)	38 040 (32.2)	42.8 (7.2)	8.8 (3.2)	34.0 (4.7)	1.53 (0.07)
1950-1959	26 826 (21.9)	31.9 (4.8)	7.0 (2.4)	24.9 (2.8)	1.39 (0.06)	26 636 (22.6)	42.9 (7.8)	8.6 (3.5)	34.3 (5.0)	1.54 (0.08)
1960-1969	18 683 (15.2)	32.4 (5.0)	7.2 (2.5)	25.3 (2.9)	1.40 (0.06)	18 618 (15.8)	44.2 (8.1)	8.9 (3.7)	35.3 (5.2)	1.56 (0.08)
1970-1985	13 575 (11.1)	33.9 (6.1)	8.0 (3.3)	25.9 (3.2)	1.40 (0.06)	10 386 (8.8)	47.1 (10.1)	10.3 (5.0)	36.8 (6.0)	1.57 (0.08)
Overall	122 678 (100)	31.8 (4.8)	7.1 (2.4)	24.8 (2.8)	1.38 (0.06)	118 025 (100)	43.0 (7.8)	8.8 (3.5)	34.2 (5.0)	1.54 (0.08)

**Table 2.** Weight, Fat Mass, Fat-Free Mass, and Height Among Girls by Adult Type 2 Diabetes Status and Birth-Cohort Group

Birth-year cohort group	10 y					13 y				
	No. (%)	Mean (SD)				No. (%)	Mean (SD)			
		Weight, kg	Fat mass, kg	Fat-free mass, kg	Height, m		Weight, kg	Fat mass, kg	Fat-free mass, kg	Height, m
**Those who developed type 2 diabetes**										
1930-1939	3223 (37.3)	30.5 (4.8)	8.0 (2.5)	22.5 (2.6)	1.35 (0.06)	3177 (37.2)	44.2 (7.7)	11.7 (4.1)	32.6 (4.3)	1.52 (0.07)
1940-1949	3375 (39.1)	32.4 (5.6)	8.9 (3.0)	23.5 (2.8)	1.37 (0.06)	3358 (39.4)	46.8 (8.6)	12.7 (4.8)	34.1 (4.5)	1.55 (0.07)
1950-1959	1440 (16.7)	33.0 (6.2)	9.1 (3.4)	23.9 (3.1)	1.38 (0.06)	1434 (16.8)	48.1 (9.4)	13.2 (5.4)	34.9 (4.7)	1.56 (0.07)
1960-1969	492 (5.7)	35.1 (7.3)	10.3 (4.3)	24.9 (3.3)	1.39 (0.06)	496 (5.8)	51.3 (10.8)	14.9 (6.4)	36.4 (5.2)	1.58 (0.07)
1970-1985	104 (1.2)	38.8 (9.4)	12.6 (5.5)	26.2 (4.3)	1.40 (0.08)	65 (0.8)	55.6 (13.7)	18.2 (8.1)	37.4 (6.3)	1.56 (0.08)
Overall	8634 (100)	32.0 (5.8)	8.7 (3.1)	23.3 (2.9)	1.37 (0.06)	8530 (100)	46.4 (8.9)	12.5 (4.9)	33.8 (4.7)	1.54 (0.07)
**Those who did not develop type 2 diabetes**										
1930-1939	25 963 (20.7)	30.1 (4.3)	7.7 (2.2)	22.4 (2.4)	1.35 (0.06)	25 893 (21.3)	43.1 (7.1)	10.9 (3.6)	32.2 (4.1)	1.52 (0.07)
1940-1949	40 008 (31.9)	31.4 (4.9)	8.2 (2.5)	23.2 (2.6)	1.37 (0.06)	39 831 (32.7)	44.7 (7.5)	11.2 (3.9)	33.5 (4.3)	1.55 (0.07)
1950-1959	27 055 (21.6)	31.6 (5.2)	8.2 (2.7)	23.4 (2.8)	1.38 (0.06)	26 987 (22.2)	45.1 (7.9)	11.1 (4.2)	34.0 (4.4)	1.56 (0.07)
1960-1969	18 800 (15.0)	32.1 (5.4)	8.3 (2.8)	23.8 (2.9)	1.39 (0.06)	18 771 (15.4)	46.1 (8.3)	11.4 (4.4)	34.7 (4.6)	1.57 (0.07)
1970-1985	13 513 (10.8)	33.8 (6.6)	9.2 (3.6)	24.6 (3.3)	1.40 (0.07)	10 155 (8.3)	48.8 (9.8)	12.9 (5.5)	35.9 (5.1)	1.58 (0.07)
Overall	125 339 (100)	31.5 (5.2)	8.2 (2.7)	23.3 (2.8)	1.37 (0.06)	121 637 (100)	45.0 (8.0)	11.3 (4.2)	33.7 (4.5)	1.55 (0.07)

### Associations of Childhood FM and Weight With T2D

Analyses were based on 269 913 children aged 10 years, spanning 7 501 643 person-years, with 21 896 T2D diagnoses (13 262 men and 8634 women) and 261 192 children aged 13 years, spanning 7 920 050 person-years, with 21 530 T2D diagnoses (13 000 men and 8530 women). On average, individuals were followed for 28 years and 30 years for men and women, respectively.

Sex-specific associations, adjusted for childhood height, between childhood FM and weight (per-kilogram increase in exposure) with T2D risk across a range of adult ages, are presented in Table 3 and Table 4 for children aged 10 and 13 years, respectively. At ages 10 and 13 years, FM was more strongly associated with higher adult T2D risk than for weight, independent of childhood height (Tables 3 and 4). These associations with adult T2D risk did not appear to vary appreciably between birth-cohort groups, were systematically stronger among girls than boys at 10 years but not at 13 years, and diminished with increasing adult age.

**Table 3.** Adjusted Hazard Ratios (95% CI) for Associations Between Fat Mass and Weight (per-Kilogram Increase) at Age 10 Years and Risk of Type 2 Diabetes Between the Ages of 30 and 70 Years by Sex, Birth-Cohort Group, and Overall

Adult age, y	Adiposity marker	Birth-year cohort group^a^					Pooled
		1930-1939	1940-1949	1950-1959	1960-1969	1970-1985	
**Boys**							
No.		29 917	438 19	29 143	19 367	13 694	NA
Cases		4588	5554	2317	684	119	
30	Fat mass	1.13 (1.08-1.20)	1.19 (1.15-1.23)	1.20 (1.15-1.24)	1.21 (1.15-1.28)	1.20 (1.13-1.27)	1.19 (1.16-1.21)
	Weight	1.06 (1.03-1.09)	1.10 (1.08-1.12)	1.11 (1.09-1.14)	1.13 (1.10-1.17)	1.14 (1.09-1.18)	1.11 (1.08-1.13)
40	Fat mass	1.11 (1.07-1.15)	1.15 (1.13-1.18)	1.16 (1.14-1.19)	1.17 (1.14-1.20)	1.19 (1.14-1.24)	1.16 (1.14-1.18)
	Weight	1.05 (1.03-1.07)	1.09 (1.07-1.10)	1.10 (1.08-1.12)	1.11 (1.10-1.13)	1.13 (1.10-1.17)	1.10 (1.07-1.12)
50	Fat mass	1.09 (1.06-1.12)	1.12 (1.10-1.13)	1.13 (1.12-1.15)	1.14 (1.10-1.17)	NA	1.12 (1.10-1.14)
	Weight	1.04 (1.03-1.06)	1.07 (1.06-1.08)	1.09 (1.08-1.10)	1.09 (1.07-1.12)	NA	1.07 (1.05-1.09)
60	Fat mass	1.06 (1.04-1.08)	1.08 (1.07-1.10)	1.10 (1.08-1.13)	NA	NA	1.08 (1.06-1.10)
	Weight	1.04 (1.02-1.05)	1.06 (1.05-1.06)	1.07 (1.06-1.09)	NA	NA	1.05 (1.04-1.07)
70	Fat mass	1.04 (1.02-1.06)	1.05 (1.03-1.07)	NA	NA	NA	1.04 (1.03-1.06)
	Weight	1.03 (1.02-1.04)	1.04 (1.03-1.05)	NA	NA	NA	1.03 (1.02-1.05)
**Girls**							
No.		29 186	43 383	28 495	19 292	13 617	NA
Cases		3223	3375	1440	492	104	
30	Fat mass	1.20 (1.14-1.27)	1.19 (1.15-1.23)	1.20 (1.15-1.25)	1.24 (1.18-1.30)	1.29 (1.22-1.37)	1.22 (1.18-1.25)
	Weight	1.10 (1.07-1.14)	1.11 (1.09-1.13)	1.13 (1.10-1.16)	1.16 (1.12-1.19)	1.20 (1.15-1.25)	1.14 (1.11-1.16)
40	Fat mass	1.17 (1.12-1.21)	1.16 (1.14-1.19)	1.17 (1.14-1.20)	1.22 (1.19-1.25)	1.28 (1.22-1.35)	1.19 (1.16-1.23)
	Weight	1.09 (1.06-1.11)	1.10 (1.08-1.12)	1.11 (1.09-1.13)	1.14 (1.12-1.17)	1.20 (1.15-1.24)	1.12 (1.10-1.15)
50	Fat mass	1.13 (1.10-1.16)	1.14 (1.12-1.16)	1.14 (1.12-1.16)	1.19 (1.15-1.23)	NA	1.15 (1.13-1.17)
	Weight	1.07 (1.06-1.09)	1.09 (1.08-1.10)	1.10 (1.08-1.11)	1.13 (1.11-1.16)	NA	1.10 (1.08-1.11)
60	Fat mass	1.10 (1.08-1.12)	1.12 (1.10-1.13)	1.11 (1.09-1.14)	NA	NA	1.11 (1.10-1.12)
	Weight	1.06 (1.05-1.07)	1.08 (1.07-1.09)	1.08 (1.06-1.10)	NA	NA	1.07 (1.06-1.08)
70	Fat mass	1.07 (1.05-1.09)	1.09 (1.07-1.12)	NA	NA	NA	1.08 (1.05-1.11)
	Weight	1.05 (1.03-1.06)	1.07 (1.06-1.08)	NA	NA	NA	1.06 (1.04-1.08)

Abbreviation: NA, not available.

^a^ Hazard ratios (95% CIs) estimated from Cox proportional-hazards models fitted within each of the 5 birth-year cohort groups, adjusting for childhood height at age 10 years. The resulting estimates were averaged using a random-effects meta-analysis approach to provide an overall estimate of the effect of fat mass and weight on type 2 diabetes risk in adulthood. Birth-year cohort groups from 1950-1959, 1960-1969, and 1970-1985 contain some cells without data; this is because the individuals within these groups were not yet old enough to provide estimates, without extrapolation, at these adult ages.

**Table 4.** Adjusted Hazard Ratios (95% CI) for Associations Between Fat Mass and Weight (per-Kilogram Increase) at Age 13 Years and Risk of Type 2 Diabetes Between the Ages of 30 and 70 Years by Sex, Birth-Cohort Group, and Overall

Adult age, y	Adiposity marker	Birth-year cohort group^a^					Pooled
		1930-1939	1940-1949	1950-1959	1960-1969	1970-1985	
**Boys**							
No.	NA	28 755	43 548	28 935	19 304	10 483	NA
Cases		4410	5508	2299	686	97	
30	Fat mass	1.13 (1.10-1.17)	1.13 (1.11-1.16)	1.14 (1.12-1.17)	1.14 (1.10-1.18)	1.09 (1.04-1.14)	1.13 (1.12-1.15)
	Weight	1.06 (1.05-1.08)	1.08 (1.06-1.09)	1.08 (1.07-1.10)	1.09 (1.07-1.11)	1.07 (1.04-1.10)	1.08 (1.07-1.08)
40	Fat mass	1.11 (1.09-1.14)	1.11 (1.10-1.13)	1.12 (1.11-1.14)	1.12 (1.10-1.14)	1.13 (1.09-1.16)	1.12 (1.11-1.13)
	Weight	1.06 (1.04-1.07)	1.07 (1.06-1.08)	1.07 (1.06-1.08)	1.08 (1.07-1.09)	1.08 (1.06-1.11)	1.07 (1.06-1.08)
50	Fat mass	1.09 (1.08-1.11)	1.09 (1.08-1.10)	1.10 (1.09-1.11)	1.11 (1.08-1.13)	NA	1.10 (1.09-1.10)
	Weight	1.05 (1.04-1.06)	1.06 (1.05-1.06)	1.07 (1.06-1.07)	1.07 (1.06-1.09)	NA	1.06 (1.05-1.07)
60	Fat mass	1.07 (1.06-1.08)	1.07 (1.07-1.08)	1.08 (1.07-1.10)	NA	NA	1.08 (1.07-1.08)
	Weight	1.05 (1.04-1.05)	1.05 (1.05-1.06)	1.06 (1.05-1.07)	NA	NA	1.05 (1.04-1.06)
70	Fat mass	1.05 (1.04-1.07)	1.06 (1.04-1.07)	NA	NA	NA	1.06 (1.05-1.06)
	Weight	1.04 (1.03-1.05)	1.04 (1.04-1.05)	NA	NA	NA	1.04 (1.04-1.05)
**Girls**							
No.	NA	29 070	43 189	28 421	19 267	10 220	NA
Cases		3177	3358	1434	496	65	
30	Fat mass	1.16 (1.12-1.19)	1.11 (1.09-1.14)	1.15 (1.12-1.18)	1.17 (1.14-1.20)	1.13 (1.07-1.19)	1.14 (1.12-1.17)
	Weight	1.08 (1.06-1.10)	1.07 (1.06-1.08)	1.10 (1.08-1.11)	1.11 (1.09-1.13)	1.09 (1.06-1.13)	1.09 (1.07-1.10)
40	Fat mass	1.13 (1.11-1.16)	1.11 (1.09-1.12)	1.13 (1.11-1.15)	1.14 (1.12-1.16)	1.17 (1.13-1.21)	1.13 (1.11-1.15)
	Weight	1.07 (1.06-1.09)	1.07 (1.06-1.08)	1.08 (1.07-1.09)	1.10 (1.08-1.11)	1.11 (1.09-1.14)	1.08 (1.07-1.10)
50	Fat mass	1.11 (1.09-1.13)	1.10 (1.09-1.11)	1.11 (1.09-1.12)	1.11 (1.09-1.13)	NA	1.10 (1.10-1.11)
	Weight	1.06 (1.05-1.07)	1.06 (1.06-1.07)	1.07 (1.06-1.08)	1.08 (1.07-1.09)	NA	1.07 (1.06-1.08)
60	Fat mass	1.08 (1.07-1.10)	1.09 (1.08-1.10)	1.08 (1.07-1.10)	NA	NA	1.09 (1.08-1.09)
	Weight	1.05 (1.05-1.06)	1.06 (1.05-1.07)	1.06 (1.05-1.07)	NA	NA	1.06 (1.05-1.06)
70	Fat mass	1.06 (1.05-1.07)	1.08 (1.07-1.09)	NA	NA	NA	1.07 (1.05-1.09)
	Weight	1.04 (1.04-1.05)	1.06 (1.05-1.06)	NA	NA	NA	1.05 (1.04-1.06)

Abbreviation: NA, not available.

^a^ Hazard ratios (95% CIs) estimated from Cox proportional-hazards models fitted within each of the 5 birth-cohort groups, adjusting for childhood height at age 13 years. The resulting estimates were averaged using a random-effects meta-analysis approach to provide an overall estimate of the effect of fat mass and weight on type 2 diabetes risk in adulthood. Birth-year cohort groups from 1950-1959, 1960-1969, and 1970-1985 contain some cells without data; this is because the individuals within these groups were not yet old enough to provide estimates, without extrapolation, at these adult ages.

Estimates pooled across birth-cohort groups (Tables 3 and 4 and Figure) suggested that for children aged 10 years, a 1-kg increase in FM was associated with an increased risk of T2D at age 50 years of 12% (HR, 1.12; 95% CI, 1.10-1.14) for boys and 15% (HR, 1.15; 95% CI, 1.13-1.17) for girls. In comparison, a 1-kg increase in weight at age 10 years was associated with an increased risk of T2D at age 50 years of 7% for boys (HR, 1.07; 95% CI, 1.05-1.09) and 10% for girls (HR, 1.10; 95% CI, 1.08-1.11) at age 50 years (Tables 3 and 4 and Figure). Pooled estimates of the associations at age 13 years (Tables 3 and 4 and Figure) suggested that a 1-kg increase in childhood FM was associated with an increased risk of T2D at age 50 years of 10% (HR, 1.10; 95% CI, 1.09-1.10) for boys and 10% (HR, 1.10; 95% CI, 1.10-1.11) for girls. In comparison, a 1-kg increase in childhood weight at age 13 years was associated with an increased risk of T2D at age 50 years of 6% for boys (HR, 1.06; 95% CI, 1.05-1.07) at age 50 years and 7% for girls (HR, 1.07; 95% CI, 1.06-1.08) at age 50 years (Tables 3 and 4 and Figure).

**Figure.**
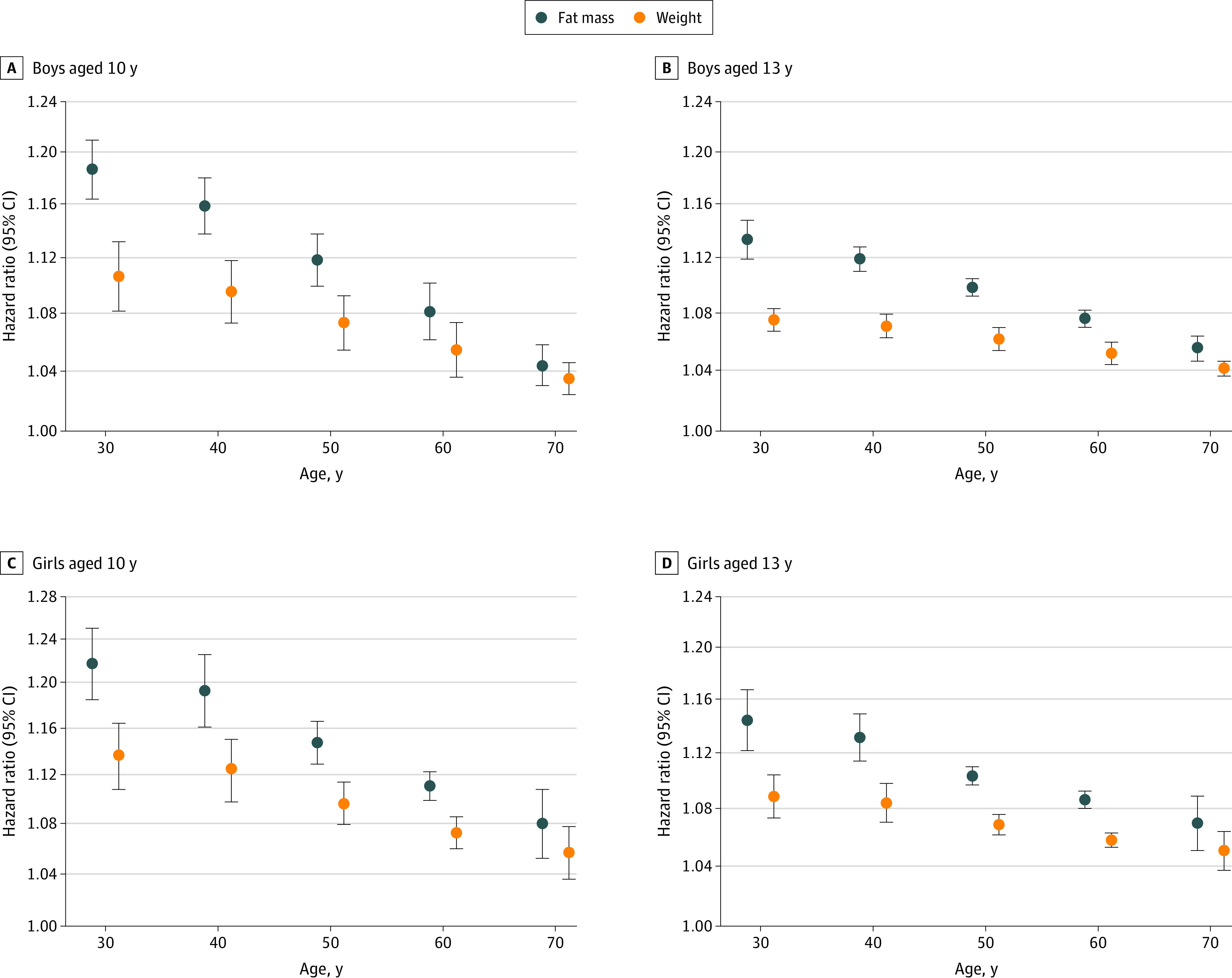
Adjusted Hazard Ratios (95% CI) for Associations Between Fat Mass and Weight (per-Kilogram Increase) at Ages 10 and 13 Years and Risk of Type 2 Diabetes Between the Ages of 30 and 70 Years by Sex Hazard ratios and 95% CIs estimated from height-adjusted Cox proportional-hazards models fitted within each of the 5 birth-cohort groups. The resulting estimates were averaged using a random-effects meta-analysis approach to provide an overall estimate of the effect of fat mass (blue dots) and weight (orange dots) on type 2 diabetes risk in adulthood for boys aged 10 years (A) and 13 years (B) and for girls aged 10 years (C) and 13 years (D). Hazard ratios on the y-axis are presented on the natural logarithmic scale. All estimates presented were significant, with *P* < .001. Whiskers represent 95% CIs. The hazard ratios and 95% CIs presented can be found in Tables 3 and 4.

Unadjusted associations of childhood FM and weight with T2D risk across a range of adult ages, presented in eTables 3 and 4 in the Supplement for children aged 10 and 13 years, respectively, show similar findings to the height-adjusted results, with stronger associations between childhood FM and adult T2D risk, than between weight and adult T2D risk (eg, for boys, a 1-kg increase in FM at age 10 years was associated with an increased risk of T2D of 10% [HR, 1.10; 95% CI, 1.09-1.11] at age 50 years vs an increased risk of 4% [HR, 1.04; 95% CI, 1.03-1.05] at age 50 years for a 1-kg increase in weight for boys at 10 years). Height-adjusted associations between childhood FM and weight with T2D risk, per SD increase in the respective adiposity markers, are presented in eTables 5 and 6 in the Supplement for children aged 10 and 13 years. Owing to the SD of FM being approximately half of that for weight (eTable 1 in the Supplement), the magnitude of the effect sizes on the SD scale for weight were greater than those for FM, particularly in more recent birth-cohort groups; however, the associated 95% CIs for childhood FM (for boys and girls at both childhood ages) and childhood weight were overlapping (eg, for boys born between 1930-1939, a 1-SD increase in FM at age 10 years was associated with an increased risk of T2D of 16% [HR, 1.16; 95% CI, 1.11-1.22] at age 50 years vs an increased risk of 18% [HR, 1.18; 95% CI, 1.11-1.26] at age 50 years for a 1-SD increase in weight for boys at 10 years) (eTables 5 and 6 in the Supplement). Sensitivity analyses, conducted to investigate the influence of elderly onset T2D on the observed FM and weight associations by repeating the Cox regression analyses, including censoring at age 60 years, did not materially affect the results (eTables 7 and 8 in the Supplement).

## Discussion

In this large cohort study, we found that after adjusting for childhood height, a 1-kg increase in childhood FM levels was more strongly associated with adult T2D risk than was childhood weight. Furthermore, height-adjusted associations for childhood weight presented per SD increase (where the SD of childhood FM was approximately half that of weight) were stronger than for childhood FM, albeit with largely overlapping 95% CIs.

The very large sample size and long period of follow-up allowed for the quantification of associations across a wide range of adult ages with high levels of precision. The quantified associations relied on the validity of the equations used to estimate childhood FM in this population. Notably, the pooled data sets used to derive the equation contained a wide range of anthropometric characteristics, which remained reasonably consistent with those of the CSHRR cohort. Moreover, the association between natural log-transformed FFM (the outcome of the prediction equation) and the predictors was unlikely to have changed over this time period, suggesting that our weight-height prediction equation worked equally well across the ranges of FM in this cohort. Although derived from UK children, the derivation data set also contained children of other European origins, which strengthened generalizability of the equation to the Danish CSHRR cohort. Furthermore, the diagnosis of adult T2D, as obtained via data linkage with national registries, was not subject to recall bias.

Previous investigations into the association between childhood adiposity and T2D risk have predominantly been based on BMI rather than on weight or FM and FFM, owing to the lack of historic cohorts with information on both childhood body composition and adult T2D diagnoses. Several studies, including earlier analyses based on the present cohort, demonstrated that increases in childhood BMI were associated with elevated T2D risks in adulthood.^21,22,23^ However, childhood BMI was not independent of height (both *r* ~ 0.3) in this cohort, which suggests that the power of 2 used to standardize weight for height was inadequate. This is problematic because childhood height has been shown previously within this cohort to be independently associated with long-term T2D risk, with shorter stature being associated with an increased risk of adult T2D risk^13^; therefore, the residual association with height is likely to have inflated estimated associations between childhood BMI and adult T2D risk. Therefore, comparing associations based on childhood BMI with the associations of FM and weight in this study would not be comparing like with like. The analysis undertaken in this study, quantifying and comparing the height-adjusted associations between childhood FM and adult T2D risk with those of weight, allowed us to return to focusing on weight itself, on which BMI is based. Our findings, which suggest positive associations between childhood weight and T2D risk, were consistent with previous studies, which found positive associations between childhood weight and risk markers of T2D in adulthood.^24,25,26^ These studies found higher childhood weight to be associated with an increase in levels of fasting insulin,^24,25^ whereas another study found an inverse association between childhood weight and attenuated insulin-stimulated glucose use in adulthood and a reduction in the ability to metabolize glucose in adulthood.^26^ Furthermore, studies comparing the associations between adult body weight and T2D to those of adult FM have reported that higher BF levels were more indicative of higher T2D risk than for body weight.^27,28^

### Implications

Body mass index has a number of limitations as a marker of childhood BF and when used to classify children with overweight or obesity. As a weight-based measure, it does not discriminate between FM and FFM, which can vary markedly in individuals with a given BMI. In this study, results suggest that when compared on a per-kilogram basis, childhood FM was more strongly associated with T2D risk in adulthood than childhood weight, suggesting that childhood FM, rather than weight, may be a more precise marker of the influence of childhood adiposity on long-term T2D risk. The presentation of a per-kilogram increase in effect sizes of childhood body composition has been favored because each of the markers are on the same scale of measurement. Although HRs were greater per SD increase in weight compared with FM outcomes per SD, the SD of weight was almost double that of FM, thereby giving rise to a potentially inflated HR for weight. We believe that in this situation, comparisons of per-kilogram increase in effect sizes of body composition markers were likely to allow for a fairer and more robust comparison of associations with prospective outcomes. The findings of this study could affect the interpretation of childhood adiposity surveillance initiatives, such as the English National Child Measurement Programme and the US National Health and Nutrition Examination Survey, in which noninvasive methods for FM assessment could be particularly useful alongside established thresholds for defining adverse childhood FM levels^29,30,31^ to aid discussions between clinicians, parents, guardians, and children. In particular, the equation used in this report could be of particular value because it relied on information already collected as part of these routine surveillance initiatives and would not require additional measurements to be collected.^16^ A Microsoft Excel–based calculator could be made available to facilitate the quick and straightforward estimation of FM in clinical and public health practice.

### Limitations

The present study had a number of limitations. Only individuals who were referred to hospital-based clinics were included in the NPR registry; therefore, the age-specific T2D incidence was likely to be underestimated in this study. However, the study data most likely provided a reasonable coverage of the occurrence of T2D in this population. A validation study, which included only 5 years of follow-up, previously estimated the sensitivity of the NPR to be 64%,^32^ suggesting that 36% of the patients treated by general practitioners were not identified in the NPR in that study. We found it probable that the completeness in capturing the patients with at least 1 diagnosis in the NPR would be higher in our study, in which individuals were followed for a median of 28 years for men and 30 years for women. Additionally, previous work based on the CSHRR cohort has demonstrated similar findings when obtaining established T2D cases via linkage to the Danish National Diabetes Register as opposed to the NPR used in this study.^21^ The prediction equation was derived from children born later (1985-2010) than those within this cohort (1930-1985), which may have affected the estimates of childhood FM. Although race/ethnicity information was not available within the CSHRR, the Danish population was predominantly of White ethnic/racial origin during the study period, which has an effect on the generalizability of the results to more ethnically diverse populations. Although the results from this study may be more robust in a single ethnic group, further work examining these associations in ethnically diverse populations would be of value in establishing the generalizability of the findings, particularly considering the established ethnic/racial differences in childhood of both FM and emerging T2D risk.^33,34,35,36,37^ The effect on FM predictions of assuming that children with this missing predictor are of White race/ethnicity was investigated and shown to be low in the original report.^16^ Direct measurements or linked information on other established risk factors of T2D in adulthood, such as adult weight and height, diet, or physical activity, were not available in this study. However, it is unlikely that the relative strengths of the associations would be altered by adjustment for other T2D risk factors, some of which may be mediators and not confounders.

## Conclusions

In this cohort study of children aged 10 and 13 years, results suggest that childhood FM was the component of weight more strongly associated with adult T2D risk; therefore, using weight-based measures to assess childhood adiposity may fail to focus on this crucial modifiable component of weight. These findings suggest that the assessment of FM could be particularly helpful for the surveillance and monitoring of childhood adiposity in the effort to reduce long-term adverse health outcomes, including T2D risk.

## Notes

Supplement. **eTable 1.** Mean (SD) of Childhood Weight, Fat Mass, Fat-Free Mass, and Height at 10 and 13 Years of Age, by Birth-Cohort Group and Overall **eTable 2.** Correlation Coefficients Between Each of the Childhood Body Composition Markers and Childhood Height, by Sex and Age **eTable 3.** Unadjusted Hazard Ratios (95% CI) for the Crude Associations Between Fat Mass and Weight (per-Kilogram Increase) at Age 10 Years and Risk of Type 2 Diabetes (T2D) Between 30 and 70 Years, by Sex, Birth-Cohort Group and Overall **eTable 4.** Unadjusted Hazard Ratios (95% CI) for the Crude Associations Between Fat Mass and Weight (per-Kilogram Increase) at Age 13 Years and Risk of Type 2 Diabetes (T2D) Between 30 and 70 Years, by Sex, Birth-Cohort Group and Overall **eTable 5.** Adjusted Hazard Ratios (95% CI) for Associations Between Fat Mass and Weight (per-SD Increase in Exposure) at Age 10 Years and Risk of Type 2 Diabetes Between 30 and 70 Years, by Sex, Birth-Cohort Group and Overall **eTable 6.** Adjusted Hazard Ratios (95% CI) for Associations Between Fat Mass and Weight (per-SD Increase in Exposure) at Age 13 Years and Risk of Type 2 Diabetes Between 30 and 70 Years, by Sex, Birth-Cohort Group and Overall **eTable 7.** Adjusted Hazard Ratios (95% CI), Censored at 60 Years of Age, for Associations Between Fat Mass and Weight (per-Kilogram Increase) at Age 10 Years and Risk of Type 2 Diabetes (T2D) Between 30 and 60 Years, by Sex, Birth-Cohort Group and Overall **eTable 8.** Adjusted Hazard Ratios (95% CI), Censored at 60 Years of Age, for Associations Between Fat Mass and Weight (per-Kilogram Increase) at Age 13 Years and Risk of Type 2 Diabetes (T2D) Between 30 and 60 Years, by Sex, Birth-Cohort Group and Overall **eFigure.** Flowchart of Available Participants and Exclusions Made in This Study
