# Supplementary material for: Association of Childhood Fat Mass and Weight With Adult-Onset Type 2 Diabetes in Denmark
Source: JAMA Netw Open. 2021 Apr 30;4(4):e218524. doi: 10.1001/jamanetworkopen.2021.8524 (PMC8087954; doi:10.1001/jamanetworkopen.2021.8524)
Supplement: Supplement. — eTable 1. Mean (SD) of Childhood Weight, Fat Mass, Fat-Free Mass, and Height at 10 and 13 Years of Age, by Birth-Cohort Group and Overall eTable 2. Correlation Coefficients Between Each of the Childhood Body Composition Markers and Childhood Height, by Sex and Age eTable 3. Unadjusted Hazard Ratios (95% CI) for the Crude Associations Between Fat Mass and Weight (per-Kilogram Increase) at Age 10 Years and Risk of Type 2 Diabetes (T2D) Between 30 and 70 Years, by Sex, Birth-Cohort Group and Overall eTable 4. Unadjusted Hazard Ratios (95% CI) for the Crude Associations Between Fat Mass and Weight (per-Kilogram Increase) at Age 13 Years and Risk of Type 2 Diabetes (T2D) Between 30 and 70 Years, by Sex, Birth-Cohort Group and Overall eTable 5. Adjusted Hazard Ratios (95% CI) for Associations Between Fat Mass and Weight (per-SD Increase in Exposure) at Age 10 Years and Risk of Type 2 Diabetes Between 30 and 70 Years, by Sex, Birth-Cohort Group and Overall eTable 6. Adjusted Hazard Ratios (95% CI) for Associations Between Fat Mass and Weight (per-SD Increase in Exposure) at Age 13 Years and Risk of Type 2 Diabetes Between 30 and 70 Years, by Sex, Birth-Cohort Group and Overall eTable 7. Adjusted Hazard Ratios (95% CI), Censored at 60 Years of Age, for Associations Between Fat Mass and Weight (per-Kilogram Increase) at Age 10 Years and Risk of Type 2 Diabetes (T2D) Between 30 and 60 Years, by Sex, Birth-Cohort Group and Overall eTable 8. Adjusted Hazard Ratios (95% CI), Censored at 60 Years of Age, for Associations Between Fat Mass and Weight (per-Kilogram Increase) at Age 13 Years and Risk of Type 2 Diabetes (T2D) Between 30 and 60 Years, by Sex, Birth-Cohort Group and Overall eFigure. Flowchart of Available Participants and Exclusions Made in This Study [file jamanetwopen-e218524-s001.pdf]

Supplementary Online Content

Hudda MT, Aarestrup J, Owen CG, et al. Association of childhood fat mass and weight with adult-onset type 2 diabetes in Denmark. *JAMA Netw Open*. 2021;4(4):e218524. doi:10.1001/jamanetworkopen.2021.8524

eTable 1. Mean (SD) of Childhood Weight, Fat Mass, Fat-Free Mass, and Height at 10 and 13 Years of Age, by Birth-Cohort Group and Overall

eTable 2. Correlation Coefficients Between Each of the Childhood Body Composition Markers and Childhood Height, by Sex and Age

eTable 3. Unadjusted Hazard Ratios (95% CI) for the Crude Associations Between Fat Mass and Weight (per-Kilogram Increase) at Age 10 Years and Risk of Type 2 Diabetes (T2D) Between 30 and 70 Years, by Sex, Birth-Cohort Group and Overall

eTable 4. Unadjusted Hazard Ratios (95% CI) for the Crude Associations Between Fat Mass and Weight (per-Kilogram Increase) at Age 13 Years and Risk of Type 2 Diabetes (T2D) Between 30 and 70 Years, by Sex, Birth-Cohort Group and Overall

eTable 5. Adjusted Hazard Ratios (95% CI) for Associations Between Fat Mass and Weight (per-SD Increase in Exposure) at Age 10 Years and Risk of Type 2 Diabetes Between 30 and 70 Years, by Sex, Birth-Cohort Group and Overall

eTable 6. Adjusted Hazard Ratios (95% CI) for Associations Between Fat Mass and Weight (per-SD Increase in Exposure) at Age 13 Years and Risk of Type 2 Diabetes Between 30 and 70 Years, by Sex, Birth-Cohort Group and Overall

eTable 7. Adjusted Hazard Ratios (95% CI), Censored at 60 Years of Age, for Associations Between Fat Mass and Weight (per-Kilogram Increase) at Age 10 Years and Risk of Type 2 Diabetes (T2D) Between 30 and 60 Years, by Sex, Birth-Cohort Group and Overall

eTable 8. Adjusted Hazard Ratios (95% CI), Censored at 60 Years of Age, for Associations Between Fat Mass and Weight (per-Kilogram Increase) at Age 13 Years and Risk of Type 2 Diabetes (T2D) Between 30 and 60 Years, by Sex, Birth-Cohort Group and Overall

eFigure. Flowchart of Available Participants and Exclusions Made in This Study

This supplementary material has been provided by the authors to give readers additional information about their work.

© 2021 Hudda MT et al. *JAMA Network Open.*

eTable 1. Mean (SD) of Childhood Weight, Fat Mass, Fat-Free Mass, and Height at 10 and 13 Years of Age, by Birth-Cohort Group and Overall

		Childhood Age									
		10 Years					13 Years				
	Birth-cohort group	N	Weight (kg)	Fat Mass (kg)	Fat-free Mass (kg)	Height (m)	N	Weight (kg)	Fat Mass (kg)	Fat-free Mass (kg)	Height (m)
**Boys**	1930-39	29,917	30.4 (4.0)	6.6 (1.8)	23.8 (2.5)	1.36 (0.06)	28,755	40.8 (6.5)	8.3 (2.9)	32.5 (4.3)	1.51 (0.07)
	1940-49	43,819	31.9 (4.5)	7.1 (2.2)	24.7 (2.6)	1.38 (0.06)	43,548	43.0 (7.3)	8.9 (3.3)	34.1 (4.7)	1.53 (0.08)
	1950-59	29,143	32.0 (4.9)	7.1 (2.4)	24.9 (2.8)	1.39 (0.06)	28,935	43.1 (7.9)	8.7 (3.7)	34.4 (5.0)	1.54 (0.08)
	1960-69	19,367	32.5 (5.1)	7.2 (2.5)	25.3 (2.9)	1.40 (0.06)	19,304	44.3 (8.2)	9.0 (3.7)	35.3 (5.3)	1.56 (0.08)
	1970-85	13,694	33.9 (6.2)	8.0 (3.3)	25.9 (3.2)	1.40 (0.06)	10,483	47.2 (10.2)	10.3 (5.1)	36.8 (6.0)	1.57 (0.08)
	Overall	135,940	31.9 (4.9)	7.1 (2.4)	24.7 (2.8)	1.38 (0.06)	131,025	43.1 (7.9)	8.9 (3.6)	34.2 (5.0)	1.54 (0.08)
**Girls**	1930-39	29,186	30.2 (4.4)	7.8 (2.2)	22.4 (2.5)	1.35 (0.06)	29,070	43.2 (7.2)	11.0 (3.6)	32.3 (4.1)	1.52 (0.07)
	1940-49	43,383	31.5 (4.9)	8.2 (2.6)	23.2 (2.7)	1.37 (0.06)	43,189	44.9 (7.6)	11.3 (4.0)	33.5 (4.3)	1.55 (0.07)
	1950-59	28,495	31.6 (5.3)	8.2 (2.8)	23.4 (2.8)	1.38 (0.06)	28,421	45.3 (8.1)	11.2 (4.3)	34.0 (4.5)	1.56 (0.07)
	1960-69	19,292	32.2 (5.5)	8.3 (2.9)	23.8 (2.9)	1.39 (0.06)	19,267	46.3 (8.4)	11.5 (4.5)	34.7 (4.6)	1.57 (0.07)
	1970-85	13,617	33.9 (6.6)	9.2 (3.6)	24.6 (3.3)	1.40 (0.07)	10,220	48.8 (9.8)	12.9 (5.5)	35.9 (5.1)	1.58 (0.07)
	Overall	133,973	31.6 (5.3)	8.2 (2.7)	23.3 (2.8)	1.37 (0.06)	130,167	45.1 (8.1)	11.4 (4.2)	33.7 (4.5)	1.55 (0.07)

© 2021 Hudda MT et al. *JAMA Network Open.*

eTable 2. Correlation Coefficients Between Each of the Childhood Body Composition Markers and Childhood Height, by Sex and Age

		**10 Years**				**13 Years**			
		Height	Fat Mass	Fat-free Mass	Weight	Height	Fat Mass	Fat-free Mass	Weight
**Boys**	Height								
	Fat Mass	0.45				0.38			
	Fat-free Mass	0.92	0.76			0.94	0.66		
	Weight	0.75	0.93	0.95		0.77	0.88	0.94	
**Girls**	Height								
	Fat Mass	0.48				0.35			
	Fat-free Mass	0.91	0.80			0.91	0.69		
	Weight	0.73	0.95	0.95		0.70	0.91	0.93	

© 2021 Hudda MT et al. *JAMA Network Open.*

eTable 3. Unadjusted Hazard Ratios (95% CI) for the Crude Associations Between Fat Mass and Weight (per-Kilogram Increase) at Age 10 Years and Risk of Type 2 Diabetes (T2D) Between 30 and 70 Years, by Sex, Birth-Cohort Group and Overall

		**Birth Cohort Group**					
**Adult Age (years)**	**Adiposity marker**	**1930-39 (29917, 4588)**	**1940-49 (43819, 5554)**	**1950-59 (29143, 2317)**	**1960-69 (19367, 684)**	**1970-85 (13694, 119)**	***Pooled***
**Boys**							
30	Fat Mass	1.12 (1.07 - 1.19)	1.17 (1.13 - 1.21)	1.17 (1.13 - 1.22)	1.20 (1.14 - 1.27)	1.18 (1.11 - 1.26)	1.17 (1.15 - 1.19)
	Weight	1.04 (1.01 - 1.07)	1.07 (1.05 - 1.09)	1.07 (1.05 - 1.10)	1.10 (1.07 - 1.14)	1.10 (1.06 - 1.15)	1.07 (1.05 - 1.09)
40	Fat Mass	1.10 (1.06 - 1.14)	1.13 (1.11 - 1.16)	1.14 (1.11 - 1.17)	1.16 (1.13 - 1.19)	1.17 (1.12 - 1.22)	1.14 (1.12 - 1.16)
	Weight	1.03 (1.01 - 1.05)	1.05 (1.04 - 1.07)	1.06 (1.04 - 1.07)	1.08 (1.06 - 1.09)	1.09 (1.06 - 1.12)	1.06 (1.04 - 1.08)
50	Fat Mass	1.08 (1.05 - 1.10)	1.10 (1.08 - 1.11)	1.11 (1.09 - 1.12)	1.12 (1.09 - 1.16)		1.10 (1.09 - 1.11)
	Weight	1.02 (1.01 - 1.04)	1.04 (1.03 - 1.04)	1.04 (1.04 - 1.05)	1.06 (1.04 - 1.07)		1.04 (1.03 - 1.05)
60	Fat Mass	1.05 (1.03 - 1.07)	1.06 (1.05 - 1.07)	1.08 (1.06 - 1.10)			1.06 (1.05 - 1.07)
	Weight	1.02 (1.01 - 1.02)	1.02 (1.01 - 1.03)	1.03 (1.02 - 1.04)			1.02 (1.01 - 1.03)
70	Fat Mass	1.03 (1.01 - 1.05)	1.03 (1.01 - 1.05)				1.03 (1.01 - 1.04)
	Weight	1.01 (1.00 - 1.02)	1.00 (1.00 - 1.01)				1.01 (1.00 - 1.01)
**Girls**		**1930-39 (29186, 3223)**	**1940-49 (43383, 3375)**	**1950-59 (28495, 1440)**	**1960-69 (19292, 492)**	**1970-85 (13617, 104)**	***Pooled***
30	Fat Mass	1.19 (1.13 - 1.25)	1.16 (1.13 - 1.20)	1.17 (1.13 - 1.23)	1.22 (1.16 - 1.28)	1.23 (1.16 - 1.30)	1.19 (1.16 - 1.21)
	Weight	1.08 (1.05 - 1.11)	1.07 (1.05 - 1.10)	1.09 (1.06 - 1.11)	1.11 (1.08 - 1.15)	1.12 (1.08 - 1.16)	1.09 (1.07 - 1.10)
40	Fat Mass	1.15 (1.11 - 1.20)	1.14 (1.11 - 1.17)	1.14 (1.11 - 1.17)	1.19 (1.16 - 1.22)	1.21 (1.15 - 1.27)	1.16 (1.14 - 1.19)
	Weight	1.06 (1.04 - 1.08)	1.06 (1.05 - 1.08)	1.07 (1.05 - 1.08)	1.09 (1.08 - 1.11)	1.10 (1.07 - 1.13)	1.08 (1.06 - 1.09)
50	Fat Mass	1.11 (1.09 - 1.14)	1.11 (1.10 - 1.13)	1.11 (1.10 - 1.13)	1.16 (1.13 - 1.19)		1.12 (1.10 - 1.14)
	Weight	1.05 (1.03 - 1.06)	1.05 (1.04 - 1.06)	1.05 (1.04 - 1.06)	1.08 (1.06 - 1.10)		1.05 (1.04 - 1.06)
60	Fat Mass	1.08 (1.06 - 1.10)	1.09 (1.08 - 1.10)	1.08 (1.06 - 1.11)			1.09 (1.08 - 1.10)
	Weight	1.03 (1.02 - 1.04)	1.04 (1.03 - 1.04)	1.03 (1.02 - 1.05)			1.03 (1.03 - 1.04)
70	Fat Mass	1.04 (1.03 - 1.06)	1.07 (1.05 - 1.09)				1.06 (1.03 - 1.08)
	Weight	1.02 (1.01 - 1.02)	1.03 (1.02 - 1.04)				1.02 (1.01 - 1.03)

FOOTNOTE: Hazard Ratios + 95% CIs estimated from Cox proportional hazards models fitted within each of the five birth cohort groups. The resulting estimates were averaged using a random-effects meta-analysis approach to provide an overall estimate of the effect of fat mass and weight on T2D risk in adulthood. Birth cohort groups from 1950-59, 1960-69 and 1970-85 contain some empty cells as individuals within these groups were not yet old enough to provide estimates, without extrapolation, at these adult ages.

© 2021 Hudda MT et al. *JAMA Network Open.*

eTable 4. Unadjusted Hazard Ratios (95% CI) for the Crude Associations Between Fat Mass and Weight (per-Kilogram Increase) at Age 13 Years and Risk of Type 2 Diabetes (T2D) Between 30 and 70 Years, by Sex, Birth-Cohort Group and Overall

		**Birth Cohort Group (N, cases)**					
**Adult Age (years)**	**Adiposity marker**	**1930-39 (28755, 4410)**	**1940-49 (43548, 5508)**	**1950-59 (28935, 2299)**	**1960-69 (19304, 686)**	**1970-85 (10483, 97)**	***Pooled***
**Boys**							
30	Fat Mass	1.13 (1.09 - 1.16)	1.13 (1.10 - 1.15)	1.14 (1.11 - 1.17)	1.14 (1.10 - 1.18)	1.08 (1.03 - 1.14)	1.13 (1.11 - 1.14)
	Weight	1.04 (1.02 - 1.06)	1.05 (1.04 - 1.06)	1.06 (1.04 - 1.07)	1.06 (1.04 - 1.09)	1.04 (1.01 - 1.07)	1.05 (1.04 - 1.06)
40	Fat Mass	1.11 (1.08 - 1.13)	1.11 (1.09 - 1.12)	1.12 (1.10 - 1.13)	1.12 (1.10 - 1.14)	1.12 (1.08 - 1.16)	1.11 (1.10 - 1.12)
	Weight	1.03 (1.02 - 1.05)	1.04 (1.03 - 1.05)	1.05 (1.04 - 1.06)	1.05 (1.05 - 1.06)	1.06 (1.04 - 1.08)	1.05 (1.04 - 1.05)
50	Fat Mass	1.09 (1.07 - 1.10)	1.09 (1.08 - 1.10)	1.10 (1.09 - 1.11)	1.10 (1.08 - 1.13)		1.09 (1.08 - 1.10)
	Weight	1.03 (1.02 - 1.04)	1.03 (1.03 - 1.04)	1.04 (1.03 - 1.04)	1.05 (1.03 - 1.06)		1.04 (1.03 - 1.04)
60	Fat Mass	1.07 (1.06 - 1.08)	1.07 (1.06 - 1.07)	1.08 (1.06 - 1.09)			1.07 (1.06 - 1.07)
	Weight	1.02 (1.02 - 1.03)	1.02 (1.02 - 1.03)	1.03 (1.02 - 1.04)			1.02 (1.02 - 1.03)
70	Fat Mass	1.05 (1.04 - 1.06)	1.05 (1.04 - 1.06)				1.05 (1.04 - 1.06)
	Weight	1.02 (1.01 - 1.02)	1.01 (1.01 - 1.02)				1.02 (1.01 - 1.02)
**Girls**		**1930-39 (29070, 3177)**	**1940-49 (43189, 3358)**	**1950-59 (28421, 1434)**	**1960-69 (19267, 496)**	**1970-85 (10220, 65)**	***Pooled***
30	Fat Mass	1.15 (1.12 - 1.19)	1.11 (1.08 - 1.13)	1.14 (1.11 - 1.17)	1.16 (1.13 - 1.20)	1.11 (1.05 - 1.17)	1.14 (1.11 - 1.16)
	Weight	1.06 (1.04 - 1.08)	1.05 (1.03 - 1.06)	1.07 (1.06 - 1.09)	1.09 (1.07 - 1.11)	1.05 (1.01 - 1.09)	1.06 (1.05 - 1.08)
40	Fat Mass	1.12 (1.10 - 1.15)	1.10 (1.08 - 1.11)	1.12 (1.10 - 1.14)	1.13 (1.12 - 1.15)	1.15 (1.11 - 1.19)	1.12 (1.11 - 1.14)
	Weight	1.05 (1.04 - 1.06)	1.04 (1.03 - 1.05)	1.06 (1.05 - 1.07)	1.07 (1.06 - 1.08)	1.07 (1.05 - 1.10)	1.06 (1.05 - 1.07)
50	Fat Mass	1.10 (1.08 - 1.12)	1.09 (1.08 - 1.10)	1.10 (1.09 - 1.11)	1.10 (1.08 - 1.12)		1.09 (1.09 - 1.10)
	Weight	1.04 (1.03 - 1.05)	1.04 (1.03 - 1.04)	1.04 (1.04 - 1.05)	1.05 (1.04 - 1.06)		1.04 (1.04 - 1.05)
60	Fat Mass	1.07 (1.06 - 1.08)	1.08 (1.07 - 1.09)	1.07 (1.06 - 1.09)			1.08 (1.07 - 1.08)
	Weight	1.03 (1.02 - 1.04)	1.03 (1.03 - 1.04)	1.03 (1.02 - 1.04)			1.03 (1.03 - 1.04)
70	Fat Mass	1.05 (1.04 - 1.06)	1.07 (1.06 - 1.08)				1.06 (1.04 - 1.08)
	Weight	1.02 (1.01 - 1.02)	1.03 (1.02 - 1.04)				1.02 (1.01 - 1.04)

FOOTNOTE: Hazard Ratios + 95% CIs estimated from Cox proportional hazards models fitted within each of the five birth cohort groups. The resulting estimates were averaged using a random-effects meta-analysis approach to provide an overall estimate of the effect of fat mass and weight on T2D risk in adulthood. Birth cohort groups from 1950-59, 1960-69 and 1970-85 contain some empty cells as individuals within these groups were not yet old enough to provide estimates, without extrapolation, at these adult ages

© 2021 Hudda MT et al. *JAMA Network Open.*

eTable 5. Adjusted Hazard Ratios (95% CI) for Associations Between Fat Mass and Weight (per-SD Increase in Exposure) at Age 10 Years and Risk of Type 2 Diabetes Between 30 and 70 Years, by Sex, Birth-Cohort Group and Overall

		Birth Cohort Group				
**Adult Age (years)**	**Adiposity marker**	**1930-39 (29917, 4588)**	**1940-49 (43819, 5554)**	**1950-59 (29143, 2317)**	**1960-69 (19367, 684)**	**1970-85 (13694, 119)**
**Boys**						
30	Fat Mass	1.26 (1.14 - 1.38)	1.46 (1.36 - 1.56)	1.54 (1.40 - 1.69)	1.62 (1.43 - 1.85)	1.81 (1.48 - 2.20)
	Weight	1.25 (1.12 - 1.39)	1.55 (1.44 - 1.68)	1.69 (1.52 - 1.88)	1.89 (1.62 - 2.21)	2.22 (1.75 - 2.82)
40	Fat Mass	1.21 (1.12 - 1.30)	1.36 (1.30 - 1.43)	1.44 (1.36 - 1.53)	1.50 (1.40 - 1.59)	1.79 (1.56 - 2.05)
	Weight	1.22 (1.12 - 1.32)	1.46 (1.37 - 1.54)	1.59 (1.48 - 1.71)	1.73 (1.59 - 1.89)	2.18 (1.82 - 2.61)
50	Fat Mass	1.16 (1.11 - 1.22)	1.27 (1.23 - 1.31)	1.35 (1.30 - 1.40)	1.38 (1.27 - 1.49)	
	Weight	1.18 (1.11 - 1.26)	1.36 (1.31 - 1.42)	1.50 (1.43 - 1.58)	1.58 (1.43 - 1.75)	
60	Fat Mass	1.12 (1.08 - 1.15)	1.19 (1.16 - 1.22)	1.27 (1.20 - 1.34)		
	Weight	1.15 (1.10 - 1.20)	1.28 (1.23 - 1.33)	1.42 (1.33 - 1.51)		
70	Fat Mass	1.07 (1.04 - 1.11)	1.11 (1.07 - 1.16)			
	Weight	1.12 (1.07 - 1.17)	1.20 (1.14 - 1.26)			
**Girls**		**1930-39 (29186, 3223)**	**1940-49 (43383, 3375)**	**1950-59 (28495, 1440)**	**1960-69 (19292, 492)**	**1970-85 (13617, 104)**
30	Fat Mass	1.50 (1.35 - 1.68)	1.55 (1.42 - 1.69)	1.67 (1.49 - 1.87)	1.87 (1.62 - 2.15)	2.55 (2.06 - 3.16)
	Weight	1.54 (1.36 - 1.75)	1.69 (1.53 - 1.85)	1.89 (1.66 - 2.15)	2.22 (1.88 - 2.64)	3.35 (2.59 - 4.34)
40	Fat Mass	1.41 (1.30 - 1.53)	1.47 (1.39 - 1.56)	1.55 (1.44 - 1.67)	1.76 (1.64 - 1.90)	2.47 (2.05 - 2.98)
	Weight	1.45 (1.32 - 1.60)	1.60 (1.49 - 1.72)	1.75 (1.60 - 1.90)	2.10 (1.90 - 2.31)	3.27 (2.59 - 4.13)
50	Fat Mass	1.32 (1.24 - 1.39)	1.40 (1.34 - 1.45)	1.45 (1.38 - 1.52)	1.66 (1.52 - 1.82)	
	Weight	1.37 (1.28 - 1.47)	1.52 (1.45 - 1.60)	1.61 (1.52 - 1.72)	1.98 (1.77 - 2.22)	
60	Fat Mass	1.23 (1.18 - 1.28)	1.33 (1.28 - 1.37)	1.35 (1.26 - 1.44)		
	Weight	1.29 (1.23 - 1.36)	1.45 (1.39 - 1.51)	1.49 (1.38 - 1.62)		
70	Fat Mass	1.15 (1.11 - 1.20)	1.26 (1.20 - 1.32)			
	Weight	1.22 (1.16 - 1.28)	1.38 (1.30 - 1.46)			

FOOTNOTE: Hazard Ratios + 95% CIs (per SD increase in adiposity marker) estimated from Cox proportional hazards models fitted within each of the five birth-cohort groups, adjusting for childhood height at age 10 years. SDs of adiposity markers at age 10 years were sex- and birth-cohort specific and can be found in Supplementary Table 1. Birth-cohort groups from 1950-59, 1960-69 and 1970-85 contain some empty cells as individuals within these groups were not yet old enough to provide estimates, without extrapolation, at these adult age

© 2021 Hudda MT et al. *JAMA Network Open.*

eTable 6. Adjusted Hazard Ratios (95% CI) for Associations Between Fat Mass and Weight (per-SD Increase in Exposure) at Age 13 Years and Risk of Type 2 Diabetes Between 30 and 70 Years, by Sex, Birth-Cohort Group and Overall

		**Birth-cohort group (N, cases)**				
**Adult Age (years)**	**Adiposity marker**	**1930-39 (28755, 4410)**	**1940-49 (43548, 5508)**	**1950-59 (28935, 2299)**	**1960-69 (19304, 686)**	**1970-85 (10483, 97)**
**Boys**						
30	Fat Mass	1.43 (1.30 - 1.56)	1.52 (1.42 - 1.62)	1.63 (1.49 - 1.79)	1.64 (1.43 - 1.87)	1.53 (1.19 - 1.97)
	Weight	1.48 (1.34 - 1.64)	1.70 (1.58 - 1.84)	1.86 (1.67 - 2.07)	2.00 (1.70 - 2.35)	1.95 (1.45 - 2.60)
40	Fat Mass	1.36 (1.27 - 1.45)	1.43 (1.37 - 1.50)	1.53 (1.45 - 1.62)	1.54 (1.45 - 1.64)	1.82 (1.54 - 2.16)
	Weight	1.43 (1.32 - 1.55)	1.61 (1.52 - 1.71)	1.77 (1.64 - 1.89)	1.88 (1.72 - 2.06)	2.21 (1.77 - 2.77)
50	Fat Mass	1.29 (1.23 - 1.35)	1.35 (1.31 - 1.39)	1.43 (1.39 - 1.48)	1.46 (1.35 - 1.57)	
	Weight	1.38 (1.30 - 1.47)	1.52 (1.46 - 1.59)	1.68 (1.60 - 1.76)	1.77 (1.60 - 1.96)	
60	Fat Mass	1.22 (1.19 - 1.26)	1.27 (1.24 - 1.31)	1.34 (1.28 - 1.41)		
	Weight	1.34 (1.28 - 1.40)	1.44 (1.39 - 1.50)	1.59 (1.50 - 1.70)		
70	Fat Mass	1.16 (1.13 - 1.20)	1.20 (1.16 - 1.25)			
	Weight	1.29 (1.23 - 1.35)	1.36 (1.30 - 1.43)			
**Girls**		**1930-39 (29070, 3177)**	**1940-49 (43189, 3358)**	**1950-59 (28421, 1434)**	**1960-69 (19267, 496)**	**1970-85 (10220, 65)**
30	Fat Mass	1.70 (1.52 - 1.90)	1.54 (1.41 - 1.68)	1.83 (1.64 - 2.04)	2.02 (1.77 - 2.30)	1.97 (1.46 - 2.66)
	Weight	1.74 (1.53 - 1.98)	1.67 (1.52 - 1.84)	2.09 (1.85 - 2.36)	2.39 (2.05 - 2.79)	2.39 (1.70 - 3.37)
40	Fat Mass	1.57 (1.44 - 1.71)	1.49 (1.41 - 1.58)	1.68 (1.57 - 1.79)	1.81 (1.69 - 1.93)	2.34 (1.95 - 2.82)
	Weight	1.64 (1.48 - 1.80)	1.64 (1.53 - 1.75)	1.91 (1.76 - 2.07)	2.14 (1.97 - 2.33)	2.90 (2.31 - 3.64)
50	Fat Mass	1.45 (1.37 - 1.54)	1.45 (1.39 - 1.50)	1.54 (1.47 - 1.60)	1.62 (1.49 - 1.76)	
	Weight	1.54 (1.43 - 1.65)	1.60 (1.52 - 1.68)	1.74 (1.65 - 1.84)	1.92 (1.74 - 2.12)	
60	Fat Mass	1.34 (1.29 - 1.39)	1.40 (1.36 - 1.44)	1.41 (1.33 - 1.50)		
	Weight	1.45 (1.37 - 1.52)	1.56 (1.50 - 1.63)	1.59 (1.48 - 1.71)		
70	Fat Mass	1.24 (1.19 - 1.28)	1.36 (1.30 - 1.42)			
	Weight	1.36 (1.29 - 1.43)	1.53 (1.45 - 1.61)			

FOOTNOTE: Hazard Ratios + 95% CIs (per-SD increase in adiposity marker) estimated from Cox proportional hazards models fitted within each of the five birth-cohort groups, adjusting for childhood height at age 13 years. SDs of adiposity markers at age 13 years were sex- and birth-cohort specific and can be found in Supplementary Table 1. Birth-cohort groups from 1950-59, 1960-69 and 1970-85 contain some empty cells as individuals within these groups were not yet old enough to provide estimates, without extrapolation, at these adult age

© 2021 Hudda MT et al. *JAMA Network Open.*

eTable 7. Adjusted Hazard Ratios (95% CI), Censored at 60 Years of Age, for Associations Between Fat Mass and Weight (per-Kilogram Increase) at Age 10 Years and Risk of Type 2 Diabetes (T2D) Between 30 and 60 Years, by Sex, Birth-Cohort Group and Overall

		**Birth Cohort Group (N, cases)**					
**Adult Age (years)**	**Adiposity marker**	**1930-39 (29917, 1262)**	**1940-49 (43819, 2649)**	**1950-59 (29143, 1991)**	**1960-69 (19367, 684)**	**1970-85 (13694, 119)**	***Pooled***
**Boys**							
30	Fat Mass	1.12 (1.01 - 1.24)	1.16 (1.11 - 1.21)	1.19 (1.14 - 1.24)	1.21 (1.15 - 1.28)	1.20 (1.13 - 1.27)	1.18 (1.15 - 1.21)
	Weight	1.07 (1.01 - 1.12)	1.10 (1.07 - 1.12)	1.11 (1.08 - 1.14)	1.13 (1.10 - 1.17)	1.14 (1.09 - 1.18)	1.11 (1.09 - 1.13)
40	Fat Mass	1.10 (1.04 - 1.17)	1.14 (1.11 - 1.17)	1.16 (1.13 - 1.19)	1.17 (1.14 - 1.20)	1.19 (1.14 - 1.24)	1.16 (1.14 - 1.18)
	Weight	1.06 (1.03 - 1.10)	1.09 (1.07 - 1.11)	1.10 (1.08 - 1.12)	1.11 (1.10 - 1.13)	1.13 (1.10 - 1.17)	1.10 (1.08 - 1.12)
50	Fat Mass	1.09 (1.06 - 1.12)	1.12 (1.11 - 1.14)	1.14 (1.12 - 1.15)	1.14 (1.10 - 1.17)		1.12 (1.11 - 1.14)
	Weight	1.06 (1.03 - 1.08)	1.08 (1.07 - 1.09)	1.09 (1.08 - 1.10)	1.09 (1.07 - 1.12)		1.08 (1.07 - 1.09)
60	Fat Mass	1.07 (1.02 - 1.13)	1.11 (1.08 - 1.14)	1.11 (1.08 - 1.14)			1.11 (1.09 - 1.12)
	Weight	1.05 (1.02 - 1.08)	1.07 (1.06 - 1.09)	1.08 (1.06 - 1.10)			1.07 (1.06 - 1.09)
**Girls**		**1930-39 (29186, 661)**	**1940-49 (43383, 1505)**	**1950-59 (28495, 1235)**	**1960-69 (19292, 492)**	**1970-85 (13617, 104)**	***Pooled***
30	Fat Mass	1.13 (1.01 - 1.25)	1.13 (1.08 - 1.18)	1.19 (1.14 - 1.24)	1.24 (1.18 - 1.30)	1.29 (1.22 - 1.37)	1.20 (1.14 - 1.26)
	Weight	1.08 (1.01 - 1.15)	1.09 (1.06 - 1.12)	1.12 (1.09 - 1.15)	1.16 (1.12 - 1.19)	1.20 (1.15 - 1.25)	1.13 (1.09 - 1.17)
40	Fat Mass	1.13 (1.06 - 1.21)	1.14 (1.11 - 1.17)	1.17 (1.14 - 1.20)	1.22 (1.19 - 1.25)	1.28 (1.22 - 1.35)	1.19 (1.14 - 1.23)
	Weight	1.09 (1.05 - 1.13)	1.10 (1.08 - 1.11)	1.11 (1.09 - 1.13)	1.14 (1.12 - 1.17)	1.20 (1.15 - 1.24)	1.13 (1.10 - 1.15)
50	Fat Mass	1.14 (1.11 - 1.18)	1.16 (1.14 - 1.18)	1.15 (1.13 - 1.17)	1.19 (1.15 - 1.23)		1.16 (1.14 - 1.17)
	Weight	1.09 (1.07 - 1.12)	1.10 (1.09 - 1.12)	1.10 (1.09 - 1.11)	1.13 (1.11 - 1.16)		1.11 (1.09 - 1.12)
60	Fat Mass	1.15 (1.09 - 1.21)	1.17 (1.14 - 1.20)	1.13 (1.10 - 1.16)			1.15 (1.12 - 1.18)
	Weight	1.10 (1.07 - 1.13)	1.11 (1.10 - 1.13)	1.09 (1.07 - 1.11)			1.10 (1.08 - 1.12)

FOOTNOTE: Survival estimates were censored at 60 years of age. Hazard Ratios + 95% CIs estimated from Cox proportional hazards models fitted within each of the five birth cohort groups, adjusting for childhood height at age 10 years. The resulting estimates were averaged using a random-effects meta-analysis approach to provide an overall estimate of the effect of fat mass and weight on T2D risk in adulthood. Birth cohort groups from 1950-59, 1960-69 and 1970-85 contain some empty cells as individuals within these groups were not yet old enough to provide estimates, without extrapolation, at these adult ages.

© 2021 Hudda MT et al. *JAMA Network Open.*

eTable 8. Adjusted Hazard Ratios (95% CI), Censored at 60 Years of Age, for Associations Between Fat Mass and Weight (per-Kilogram Increase) at Age 13 Years and Risk of Type 2 Diabetes (T2D) Between 30 and 60 Years, by Sex, Birth-Cohort Group and Overall

		**Birth Cohort Group (N, cases)**					
**Adult Age (years)**	**Adiposity marker**	**1930-39 (28755, 1207)**	**1940-49 (43548, 2627)**	**1950-59 (28935, 1975)**	**1960-69 (19304, 686)**	**1970-85 (10483, 97)**	***Pooled***
**Boys**							
30	Fat Mass	1.12 (1.06 - 1.19)	1.10 (1.08 - 1.13)	1.14 (1.11 - 1.17)	1.14 (1.10 - 1.18)	1.09 (1.04 - 1.14)	1.12 (1.10 - 1.14)
	Weight	1.06 (1.03 - 1.09)	1.07 (1.05 - 1.08)	1.08 (1.06 - 1.09)	1.09 (1.07 - 1.11)	1.07 (1.04 - 1.10)	1.07 (1.07 - 1.08)
40	Fat Mass	1.11 (1.07 - 1.15)	1.10 (1.08 - 1.12)	1.12 (1.10 - 1.14)	1.12 (1.10 - 1.14)	1.13 (1.09 - 1.16)	1.12 (1.11 - 1.13)
	Weight	1.06 (1.04 - 1.08)	1.07 (1.06 - 1.08)	1.07 (1.06 - 1.08)	1.08 (1.07 - 1.09)	1.08 (1.06 - 1.11)	1.07 (1.07 - 1.08)
50	Fat Mass	1.09 (1.07 - 1.11)	1.10 (1.09 - 1.11)	1.11 (1.09 - 1.12)	1.11 (1.08 - 1.13)		1.10 (1.10 - 1.11)
	Weight	1.06 (1.05 - 1.07)	1.07 (1.06 - 1.07)	1.07 (1.06 - 1.08)	1.07 (1.06 - 1.09)		1.07 (1.06 - 1.07)
60	Fat Mass	1.08 (1.05 - 1.11)	1.10 (1.08 - 1.12)	1.09 (1.07 - 1.11)			1.09 (1.08 - 1.10)
	Weight	1.06 (1.04 - 1.08)	1.07 (1.06 - 1.08)	1.07 (1.06 - 1.08)			1.06 (1.06 - 1.07)
**Girls**		**1930-39 (29070, 653)**	**1940-49 (43189, 1496)**	**1950-59 (28421, 1232)**	**1960-69 (19267, 496)**	**1970-85 (10220, 65)**	***Pooled***
30	Fat Mass	1.09 (1.02 - 1.17)	1.06 (1.03 - 1.10)	1.14 (1.11 - 1.17)	1.17 (1.14 - 1.20)	1.13 (1.07 - 1.19)	1.12 (1.08 - 1.17)
	Weight	1.05 (1.02 - 1.09)	1.05 (1.03 - 1.06)	1.09 (1.07 - 1.11)	1.11 (1.09 - 1.13)	1.09 (1.06 - 1.13)	1.08 (1.05 - 1.11)
40	Fat Mass	1.10 (1.06 - 1.15)	1.08 (1.07 - 1.10)	1.13 (1.11 - 1.14)	1.14 (1.12 - 1.16)	1.17 (1.13 - 1.21)	1.12 (1.10 - 1.15)
	Weight	1.07 (1.04 - 1.09)	1.06 (1.05 - 1.07)	1.08 (1.07 - 1.09)	1.10 (1.08 - 1.11)	1.11 (1.09 - 1.14)	1.08 (1.07 - 1.10)
50	Fat Mass	1.11 (1.09 - 1.14)	1.11 (1.09 - 1.12)	1.11 (1.10 - 1.12)	1.11 (1.09 - 1.13)		1.11 (1.10 - 1.12)
	Weight	1.08 (1.06 - 1.09)	1.07 (1.06 - 1.08)	1.07 (1.07 - 1.08)	1.08 (1.07 - 1.09)		1.07 (1.07 - 1.08)
60	Fat Mass	1.13 (1.09 - 1.16)	1.13 (1.11 - 1.15)	1.09 (1.07 - 1.11)			1.11 (1.09 - 1.14)
	Weight	1.09 (1.07 - 1.11)	1.09 (1.08 - 1.10)	1.06 (1.05 - 1.08)			1.08 (1.06 - 1.10)

FOOTNOTE: Survival estimates were censored at 60 years of age. Hazard Ratios + 95% CIs estimated from Cox proportional hazards models fitted within each of the five birth cohort groups, adjusting for childhood height at age 13 years. The resulting estimates were averaged using a random-effects meta-analysis approach to provide an overall estimate of the effect of fat mass and weight on T2D risk in adulthood. Birth cohort groups from 1950-59, 1960-69 and 1970-85 contain some empty cells as individuals within these groups were not yet old enough to provide estimates, without extrapolation, at these adult ages

© 2021 Hudda MT et al. *JAMA Network Open.*

eFigure. Flowchart of Available Participants and Exclusions Made in This Study

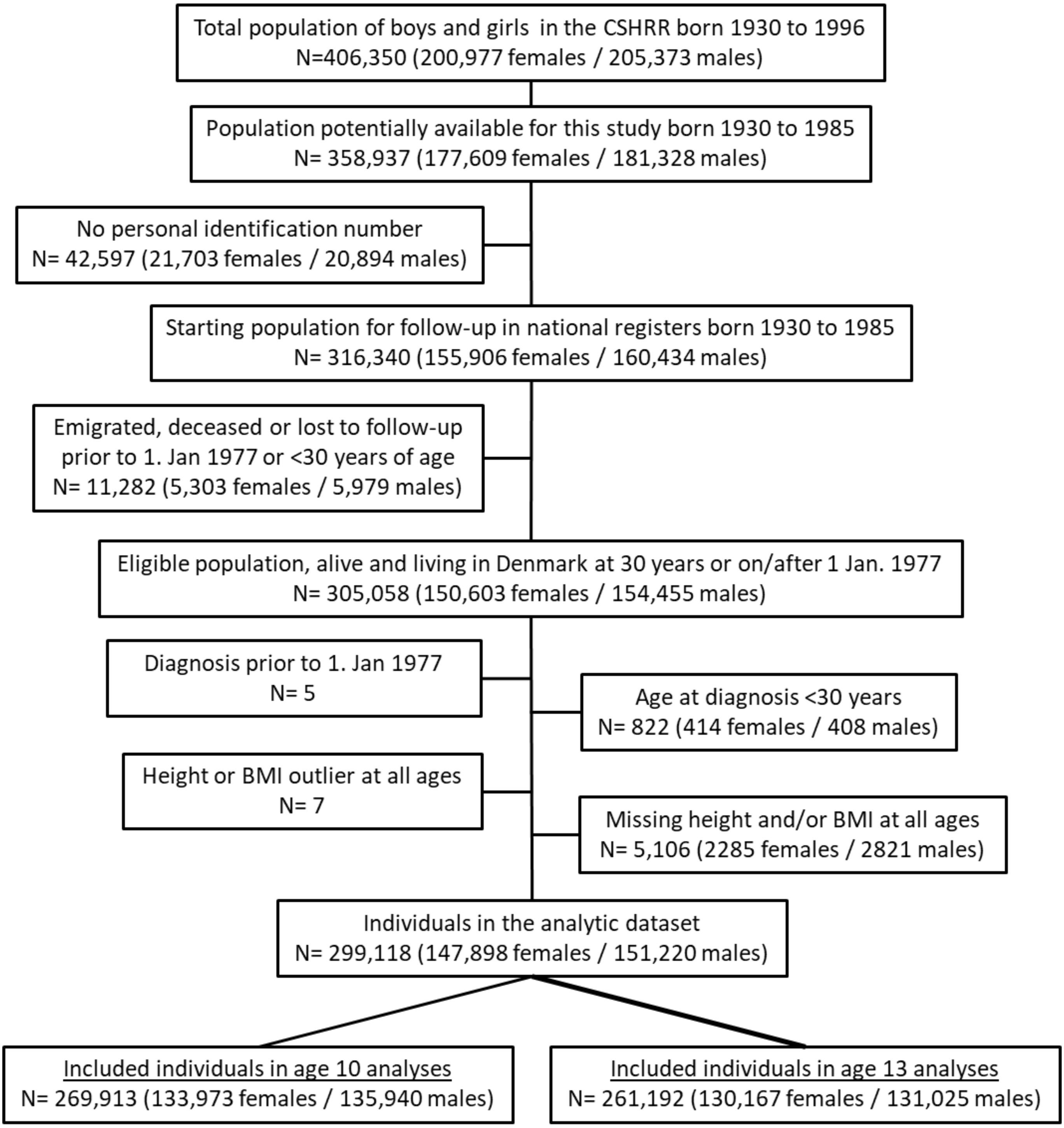

© 2021 Hudda MT et al. *JAMA Network Open.*
